# Plant-derived food bioactives in bone health: from multi-targeted roles to molecular mechanisms in osteoporosis

**DOI:** 10.3389/fnut.2025.1730053

**Published:** 2026-01-12

**Authors:** Yi Zhang, Lijuan Xu, Haofeng Xu, Yiran Zhang, Linxiao Wang, Shasha Jiang, Yan Sun, Yan Yu

**Affiliations:** 1 Department of Clinical Laboratory, Honghui Hospital, Xi'an Jiaotong University, Xi'an, China; 2 Honghui Hospital, Xi'an Jiaotong University, Xi'an, China

**Keywords:** bone formation, bone resorption, carotenoids, food bioactives, osteoporosis, polyphenols, saponins

## Abstract

Osteoporosis (OP) is a metabolic bone disease characterized by reduced bone mass and deterioration of bone microstructure. Current pharmacological treatments are often associated with significant side effects and poor patient compliance. In recent years, food bioactives—such as polyphenols, carotenoids, and saponins—have attracted growing interest for their multi-target and low-toxicity profiles in the prevention and management of OP. This review systematically elaborates the protective roles and underlying molecular mechanisms of these compounds against OP. Polyphenols exert beneficial effects through antioxidant, anti-inflammatory, and bone metabolism-regulating properties, as well as via modulation of the gut–bone axis. Their mechanisms involve key signaling pathways, including PI3K/Akt, sirtuin 1 (SIRT1)/forkhead box O3a (FOXO3a), Hippo/YAP, reactive oxygen species (ROS)/HIF-1α, and Wnt/β-catenin. Carotenoids, which are potent antioxidants, contribute to a reduced risk of OP by alleviating oxidative stress and cellular senescence, including the senescence-associated secretory phenotype (SASP). Saponins regulate bone remodeling bidirectionally through pathways such as PI3K/Akt/mTOR, bone morphogenetic protein 2 (BMP-2)/runt-related transcription factor 2 (Runx2), and RANKL/osteoprotegerin (OPG). They also inhibit NF-κB/mitogen-activated protein kinase (MAPK) signaling and downregulate osteoclast-related transcription factors, including c-Fos and NFATc1. Given their efficacy and safety, food bioactives represent a valuable source of novel nutraceuticals for bone health.

## 1 Introduction

Osteoporosis (OP) is a systemic skeletal disease characterized by micro-architectural deterioration of bone tissue and reduced bone mineral density (BMD), resulting in increased skeletal fragility and fracture susceptibility (1). Osteoporosis represents the most common bone disorder. Global data indicate that the incidence of osteoporotic fracture remains high, with a worldwide prevalence of 19.7% (10.6% in men and 24.8% in women) (2). The burden is most pronounced in regions undergoing rapid demographic aging. A 2024 survey, for example, reported an overall prevalence in China of 18.2% (11.5% male, 23.4% female), rising steadily with age—a stark illustration of the scale of the problem in the world's most populous nation (3). Similarly, 2023 figures from developed Asia-Pacific economies confirm that OP is already common across the region (4). With the global population continuing to age, this epidemiological trend is set to persist. The condition predominates in the elderly and in post-menopausal women, in whom the precipitous decline in estrogen leads to hormonal imbalance and accelerated bone loss (5, 6). Often referred to as a “silent disease,” OP is typically asymptomatic until a fragility fracture occurs—even after minor trauma or a fall from standing height. Such fractures are associated with considerable morbidity and increased mortality (7). Although genetic factors modulate mid-life BMD, age and modifiable lifestyle determinants now exert the dominant influence (8).

OP requires therapeutic intervention to upregulate BMD through pharmacological agents and/or natural compounds. Conventional treatments for bone diseases, however, are often associated with adverse effects. For instance, a meta-analysis found that patients receiving denosumab at osteoporotic doses experienced a higher incidence of serious infective adverse events (9). Current anti-osteoporotic medications consist of antiresorptive agents—such as bisphosphonates, cathepsin K (CTSK) inhibitors, and selective estrogen receptor modulators (10)—as well as vitamin D_3_, the parathyroid hormone analog teriparatide, and the receptor activator of nuclear factor-κB (NF-κB) ligand (RANKL) monoclonal antibody denosumab (11). These treatments are linked to complications including hypercalcaemia, hypercalciuria, vasomotor symptoms, breast tenderness, thromboembolic events, and an elevated risk of endometrial or breast carcinoma (12). In contrast, plant-derived compounds have demonstrated the potential to improve skeletal health without the adverse effects commonly associated with synthetic pharmaceuticals (13, 14).

In recent years, plant-derived bioactives have emerged as rational candidates for promoting skeletal health (15). For centuries, functional molecules from edible-medicinal plants have been used to improve bone quality, and their use as dietary supplements for the prevention of osteoporosis is now considered feasible (16). Parallel advances in genomics, proteomics, transcriptomics and metabolomics have created an experimentally grounded route by which these botanical molecules can be translated into mainstream drug development. A substantial proportion exhibit measurable antioxidant, anti-inflammatory, antimicrobial and anti-carcinogenic activities (17–19). Mechanistically, they suppress bone loss, preserve micro-architecture and reduce fragility by attenuating oxidative stress, modulating autophagy, dampening inflammation, reshaping the gut microbiota and adjusting estrogen levels, all with a comparatively mild side-effect profile (16). Specifically, they can tilt the RANKL/osteoprotegerin (OPG) axis in favor of osteoblast survival—down-regulating RANKL while up-regulating OPG—thereby delivering anti-osteoporotic efficacy (20, 21). Nevertheless, concentration- and structure-dependent issues of toxicity, drug–drug interaction and physicochemical stability mandate full pre-clinical and clinical evaluation before any botanical bioactive can be recommended routinely (17, 18). Despite these challenges, such natural compounds remain a highly attractive alternative to synthetic drugs whose adverse-event burden is often appreciably higher (17). Among the array of plant-derived compounds, the polyphenols, carotenoids and saponins have emerged as the most persuasive agents. They beneficially re-programme bone metabolism by curbing resorption, conserving bone-mineral density and blocking osteoclast differentiation (22–24). Consequently, we systematically reviewed the roles and mechanisms of the currently most promising plant-derived bioactive substances—polyphenols, carotenoids, and saponins—in osteoporosis, providing a potential, multi-targeted, and low-toxicity alternative to conventional therapeutic approaches for the management of osteoporosis.

## 2 Literature search strategy

A systematic literature search was conducted to identify all relevant studies on plant-derived food bioactives (polyphenols, carotenoids, and saponins) and their role in osteoporosis (OP). The primary database searched was PubMed/MEDLINE. The search strategy was built using Medical Subject Headings (MeSH) terms and key free-text words to ensure a comprehensive retrieval of records. The core search concepts included:

Bioactive Compounds: “Polyphenols” [Mesh] OR “Carotenoids” [Mesh] OR “Saponins” [Mesh] OR “Phytochemicals” [Mesh]

Disease/Condition: “Osteoporosis” [Mesh] OR “Osteogenesis” [Mesh] OR “Bone Resorption” [Mesh] OR “Osteoclasts” [Mesh] OR “Osteoblasts” [Mesh].

These concepts were combined using the Boolean operator “AND.” The search was supplemented with free-text terms such as “bone health,” “bone mineral density,” “osteogenic differentiation,” and “osteoclastogenesis” to capture additional relevant studies. The search was limited to articles published from database inception until May 2025 and to those written in English. The detailed PubMed search strategy is provided as follows: (“Polyphenols” [Mesh] OR “Carotenoids” [Mesh] OR “Saponins” [Mesh] OR “Phytochemicals” [Mesh]) AND (“Osteoporosis” [Mesh] OR “Osteogenesis” [Mesh] OR “Bone Resorption” [Mesh] OR “Osteoclasts” [Mesh] OR “Osteoblasts” [Mesh] OR “bone health” OR “bone mineral density”).

The retrieved records were initially screened by title and abstract. Studies that investigated the molecular mechanisms, efficacy, or biological effects of the specified bioactives on bone metabolism or OP models were selected for full-text review. The full texts of these articles were then assessed for eligibility based on the inclusion and exclusion criteria. The reference lists of key review articles were also manually examined to identify any additional relevant publications that might have been missed in the electronic search.

## 3 Pathogenesis of OP

Bone is a highly dynamic mineralised connective tissue whose structural integrity and functional capacity rely on a tight functional coupling between the mineralised organic matrix and its resident, lineage-committed cells (25). Skeletal homeostasis is maintained through a balance between osteoblast-mediated bone formation and osteoclast-mediated resorption. This critical balance is supplied by osteoprogenitor cells derived from bone marrow mesenchymal stem cells (BM-MSCs) (26).

### 3.1 Main pathways of bone formation

Osteoblasts drive bone matrix synthesis and subsequent mineralisation, with tissue-non-specific alkaline phosphatase (ALP) and osteocalcin (OCN) serving as key functional biomarkers of osteogenic activity (27, 28). The transcription factors runt-related transcription factor 2 (Runx2) and osterix act as master regulators that initiate and stabilize osteoblastic commitment of BM-MSCs, while preserving their self-renewal and multilineage potential—essential for lifelong bone remodeling and repair (29, 30). Osteoblast differentiation and function are regulated by evolutionarily conserved signaling pathways, most notably the bone morphogenetic protein (BMP) and canonical Wnt/β-catenin cascades (29, 31). These pathways converge on Runx2 and osterix to ensure coordinated expression of bone matrix proteins and orchestrated mineralisation (17).

#### 3.1.1 BMP pathway

BMP signaling centers on two converging arms: a canonical route in which activated type-I receptors phosphorylate Smad1/5/8, forming a Smad4-bound trimer that translocates to the nucleus and, with Runx2/Osterix, switches on osteogenic genes (OCN, ALP); and a non-canonical arm that engages p38 and extracellular signal-regulated kinase (ERK) to drive MSC proliferation and osteoblast survival. Together they commit MSCs to the osteoblastic lineage, hasten maturation and promote matrix mineralisation (32).

#### 3.1.2 Wnt/β-catenin pathway

Wnt/β-catenin signaling is the master anabolic pathway for bone formation: it blocks GSK-3β-driven β-catenin degradation, allowing β-catenin to accumulate, enter the nucleus and partner with Runx2/Osterix to switch on osteogenic genes (OCN, ALP, and COL1A1) while repressing adipogenic fate, thereby boosting matrix production and mineralisation to sustain skeletal development and post-natal bone homeostasis (33, 34).

#### 3.1.3 Phosphatidylinositol 3-kinase/protein kinase B (PI3K/Akt)/mTOR axis

The PI3K/Akt/mTOR axis is a master regulator of skeletal homeostasis. Akt-driven signaling steers BM-MSCs into osteoblasts, amplifying Runx2, Osterix, ALP and OCN expression, while boosting proliferation, matrix output and mineralisation. Simultaneously, it curbs osteoclastogenesis by dampening NF-κB/mitogen-activated protein kinase (MAPK) cues, raising OPG and lowering RANKL, thereby restraining resorption. mTOR-dependent autophagy shields osteoblasts from oxidative stress and apoptosis under high-glucose insults, and cross-talk with AMPK fine-tunes the whole network to preserve bone mass (35, 36).

### 3.2 Main pathways of bone resorption

In contrast to the anabolic function of osteoblasts, multinucleated osteoclasts mediate bone resorption through focal dissolution of the mineralised matrix. Excessive osteoclastic activity is a primary mechanism underlying net bone loss. Mature osteoclasts attach to the bone surface and establish a sealed microenvironment into which they secrete protons to solubilise hydroxyapatite, along with CTSK and matrix metalloproteinases that degrade the organic component of bone (37–39).

### 3.2.1 RANKL/RANK/OPG axis

Osteoclast differentiation, activity, and survival are predominantly regulated by RANKL–receptor activator of nuclear factor-κB (RANK)–OPG axis and macrophage colony-stimulating factor (37). Osteoblast-lineage cells produce both membrane-bound and soluble RANKL. Binding of RANKL to RANK on osteoclast precursors promotes their fusion, activation, and extended survival (40). Concurrently, osteoblasts secrete OPG, a decoy receptor that competitively binds RANKL, thereby inhibiting osteoclastogenesis (37). RANKL–RANK signaling further enhances bone resorption by stimulating the production of pro-inflammatory cytokines—such as tumor necrosis factor-α (TNF-α), interleukin-1 (IL-1), and IL-7 (41, 42)—and by upregulating key transcription factors including c-Fos and nuclear factor of activated T cells c1 (NFATc1), which are central to terminal osteoclast differentiation (43, 44). The RANKL/OPG ratio serves as a critical molecular regulator determining the overall rate of bone turnover (1). Osteoblast-lineage cells and osteoclasts engage in continuous bidirectional communication, which calibrates the rate and extent of bone remodeling. This coupling represents a fundamental mechanism in the maintenance of skeletal homeostasis (1, 40, 45). When osteoclastic resorption persistently exceeds osteoblastic bone formation, a cumulative negative balance develops, leading to the initiation and progression of OP (46, 47).

### 3.3 Disruption of homeostasis in OP: key pathological processes

Disruption of skeletal homeostasis is driven by three interrelated pathological processes: aging-related microenvironmental alterations, defective autophagy, and dysregulated apoptosis. Firstly, with advancing age, senescent bone marrow-derived BM-MSCs accumulate and acquire a senescence-associated secretory phenotype (SASP)—a pro-inflammatory secretome marked by the elevated release of IL-6, IL-1β, and TNF-α (48). SASP not only intrinsically impairs osteogenic differentiation but also acts in a paracrine manner to NF-κB signaling in adjacent healthy BM-MSCs. This induces “secondary senescence,” perpetuating a self-reinforcing cycle of impaired bone formation that contributes to age-related OP (48). Autophagic activity is significantly diminished in osteoblasts and BM-MSCs from osteoporotic patients. This reduction compromises the clearance of dysfunctional organelles and extracellular matrix components, thereby amplifying catabolic signaling and accelerating bone loss (49). Furthermore, chronic NF-κB activation and elevated RANKL levels create a pro-apoptotic microenvironment: osteoblast survival is compromised, while caspase-mediated osteoclast apoptosis is suppressed (50). The resulting “double hit”—comprising attenuated bone formation and sustained resorption—severely disrupts remodeling equilibrium and culminates in net bone loss (51–53).

### 3.4 The gut-bone axis

**Figure 1:**
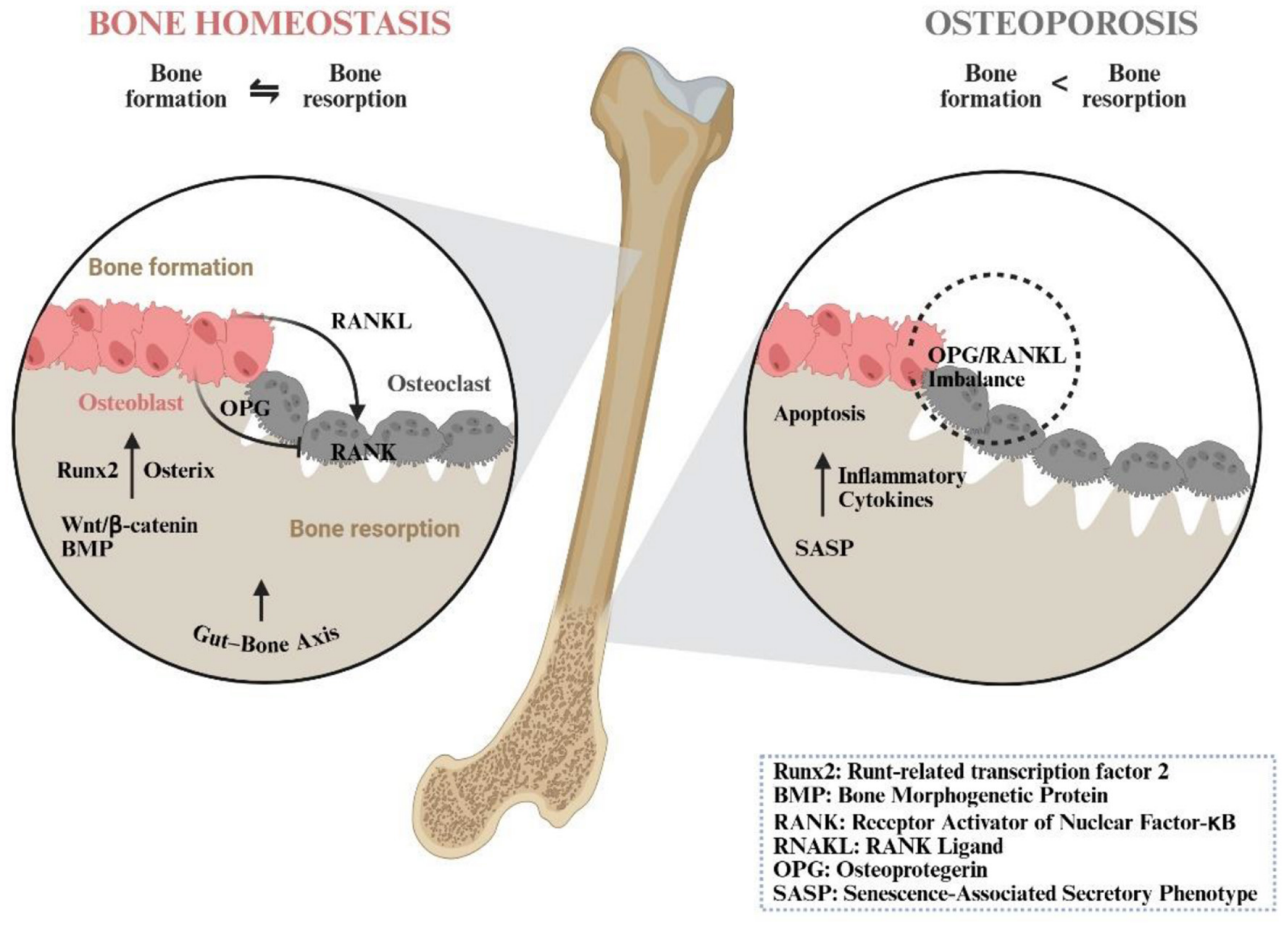
The imbalance between bone formation and bone resorption leads to OP. Bone metabolic homeostasis is fundamental to skeletal health. Osteoblast activity is principally regulated by the BMP and Wnt/β-catenin pathways. These signaling cascades converge to activate key transcription factors—such as Runx2 and osterix—which ultimately coordinate bone matrix synthesis and mineralisation. Osteoclastogenesis is primarily governed by the RANKL–RANK–OPG axis: RANKL, secreted by osteoblasts, to RANK on osteoclast precursors promotes osteoclast differentiation and survival. OPG, also produced by osteoblasts, acts as a decoy receptor that competitively binds RANKL, thereby inhibiting osteoclast formation and activity.Disruption of bone metabolic equilibrium is implicated in multiple pathological processes. An elevated RANKL/OPG ratio—a key indicator of bone turnover—drives bone loss. Furthermore, senescent BM-MSCs adopt a SASP with pro-inflammatory properties. This inflammatory microenvironment not only directly impairs osteogenic differentiation but also disrupts fundamental physiological processes such as apoptosis. Additionally, gut microbiota and their metabolites indirectly modulate bone remodeling via the gut–bone axis, further linking systemic and local regulation of bone metabolism.

The “gut–bone axis” has recently emerged as a pivotal regulator of skeletal metabolism. Intestinal microbiota and their bioactive metabolites engage in systemic cross-talk that indirectly modulates bone remodeling (54). By enhancing systemic anti-inflammatory pathways, they mitigate pro-inflammatory signaling within the bone microenvironment, thereby helping to preserve skeletal integrity (55). This paradigm broadens current understanding of metabolic bone regulation and identifies modulation of microbial composition and function as a promising therapeutic strategy for OP (56) (Figure 1).

## 4 The anti-OP effects of plant-derived bioactives

### 4.1 The therapeutic potential of polyphenols in osteoporosis: antioxidant, anti-inflammatory, and pro-osteogenic actions

Polyphenols, which are natural bioactive compounds found in various medicinal plants, aromatic herbs, foods, and beverages, possess diverse biological properties, including antioxidant, anti-inflammatory, anti-insulin resistance, and osteogenic activities. They beneficially modulate bone metabolism by attenuating bone resorption, preserving bone density, and inhibiting osteoclast differentiation. Moreover, polyphenols can concurrently target multiple molecular markers within various signaling pathways (22, 57). Beyond these actions, polyphenols induce osteoclast apoptosis and inhibit RANKL-induced osteoclastogenesis and reactive oxygen species (ROS) production (58, 59). They also stimulate the synthesis of bone morphogenetic protein 2 (BMP-2) and reduce the secretion of bone-resorptive cytokines such as TNF-α and IL-6 (60, 61).

#### 4.1.1 Modulation of bone metabolism

Polyphenols possess antioxidant, anti-inflammatory, and bone metabolism-regulating properties. Their combination with hydrogels can enhance bone tissue repair (62). Such combinations not only regulate local bone metabolism and oxidative stress but also provide mechanical support and tissue adhesion, thereby facilitating osteoporotic bone regeneration (62). Although plant-derived polyphenols inhibit osteoclast differentiation by reducing ROS generation, their bioavailability is limited due to poor intestinal absorption (63). In contrast, 4-hydroxyphenylacetic acid (4-HPA)—a microbial metabolite of polyphenols produced by the gut microbiota—effectively suppresses osteoclast differentiation and function. It downregulates key osteoclast-specific genes, including NFATc1, vacuolar-type proton ATPase subunit d2 (Atp6v0d2), matrix metallopeptidase 9, CTSK, acid phosphatase 5, and c-Fos (63). The underlying mechanism involves 4-HPA-mediated activation of nuclear factor erythroid 2-related factor 2 (Nrf2), which reduces ROS accumulation and consequently inhibits the NF-κB and MAPK signaling pathways (63). Furthermore, polyphenols enhance the expression of osteogenic markers such as Runx2, ALP, OCN, and osterix. They also inhibit the secretion of cytokines and interleukins from senescent BM-MSCs, which otherwise promote senescence in young BM-MSCs. By preserving the pool of young BM-MSCs—which are essential for osteoblast differentiation and new bone formation—polyphenols play a crucial role in mitigating OP and maintaining skeletal integrity (48).

#### 4.1.2 Resveratrol: a prototypical polyphenol with extensive evidence

Resveratrol has been one of the most extensively investigated natural polyphenols in recent years. It exerts multi-target effects and demonstrates considerable therapeutic potential in delaying aging, cardioprotection, and anticancer activities (64). Dietary resveratrol at appropriate doses is generally regarded as clinically safe and non-toxic (65). Numerous studies indicate its efficacy against various forms of OP, including those associated with chronic kidney disease (66, 67), breast cancer treatment (68), high-altitude hypoxia (69), and androgen deficiency (70). Furthermore, Qu et al. (71) reported that resveratrol can prevent or reverse rosiglitazone-induced OP in patients with type 2 diabetes (71). The combination of resveratrol and equol may favorably modulate bone turnover markers and BMD, suggesting a potential strategy for preventing age-related bone loss in post-menopausal women (72). Resveratrol significantly enhances the proliferative capacity of BM-MSCs in osteoporotic patients and plays a crucial role in regulating their pluripotency, osteogenic differentiation, and adipogenic differentiation—mechanisms integral to its anti-osteoporotic effects (73). Along with other polyphenols such as curcumin and quercetin, resveratrol positively modulates bone metabolism and osteoclast-related disorders (74). Additional studies indicated that resveratrol can counteract iron-overload-induced OP via its antioxidant properties (75). Nevertheless, its efficacy is not universal across all forms of OP. For instance, Zama et al. observed that it did not enhance implant-related bone repair in ovariectomised rats with OP (76).

#### 4.1.3 Modulation of the gut-bone axis

Dysbiosis of the gut microbiota represents a promising early clinical indicator for an elevated risk of OP (77). Polyphenols act as modulators and inducers within the gut–bone–immune axis by enhancing the abundance and functional activity of gut microbial communities (77). The metabolic profile of resveratrol may serve as a potential therapeutic biomarker for evaluating both its efficacy and associated changes in gut microbiota composition (78). Tea-polyphenol intervention also reshapes the gut microbial community and the serum metabolite profile in osteoporotic mice (79). Furthermore, polyphenols promote the proliferation of probiotic species in the gut, which contributes to reduced bone resorption and increased BMD, thereby exerting anti-osteoporotic effects (80).

#### 4.1.4 Dietary sources and clinical translation

A diet rich in polyphenols modulates the gut and oral microbiota, influencing the gut–bone axis and supporting the potential of resveratrol as both a preventive and adjunctive therapeutic agent for primary and secondary OP (78). Flavonoids, a subclass of polyphenols, are recommended for bone health maintenance due to their antioxidant, anti-inflammatory, and osteogenic properties. Higher dietary intake of flavones and flavanones is significantly associated with reduced bone loss at the femoral neck, though not in the lumbar spine (81). Therapeutically, polyphenols are metabolized into bioactive compounds that inhibit inflammatory factors, enhance gut barrier integrity, and modulate immune responses, collectively suppressing bone loss and osteoclastogenesis (82). Resveratrol, in particular, holds considerable promise as a dietary supplement or pharmaceutical agent for the clinical management of OP (83). For post-menopausal women without overt OP, twice-daily supplementation with 75 mg of resveratrol may attenuate bone loss in fracture-prone sites such as the lumbar spine and femoral neck (84). Moreover, several clinical trials have demonstrated that higher polyphenol intake raises circulating OCN and ALP and ameliorates osteopenia in post-menopausal women (85–87). Additionally, a study by Asfha et al. (88) demonstrated that teff (an Ethiopian grain)-derived biscuits containing eight flavonoid polyphenols may prevent OP through RANKL binding and interaction with key osteoclastic signaling sites (88). Freeze-dried strawberry powder, rich in polyphenols, has been shown to elevate levels of insulin-like growth factor-1, a bone-forming hormone, in post-menopausal women with pre-hypertension or stage 1 hypertension (89). Trifolirhizin also emerges as a novel candidate for the treatment of senile and post-menopausal OP (90).

### 4.2 Carotenoids as potent antioxidants and bone-protective agents

ROS-induced oxidative stress is closely associated with an increased risk of OP. Certain dietary antioxidants can help mitigate this oxidative damage (91). Notably, studies have shown that plasma retinol and all detected carotenoid levels are consistently lower in women with OP compared to healthy controls (92, 93). As potent antioxidants, carotenoids also serve as a dietary source of vitamin A and are believed to play a crucial role in disease prevention and health maintenance (94).

#### 4.2.1 Modulation of bone metabolism

Carotenoids directly engage with, and modulate, bone-metabolic homeostasis through several routes. Lycopene is among the most intensively studied. Over two decades ago, Kim et al. first reported that lycopene stimulates the proliferation and differentiation of human osteoblasts, implying its potential role in OP prevention (95). Subsequent studies in ovariectomised rats show that lycopene preserves osteoblast function, restrains resorption and, by curbing excessive turnover, restores both mechanical strength and trabecular microarchitecture (96, 97). A clinical study further demonstrated that lycopene supplementation significantly reduces oxidative stress parameters and the bone resorption marker N-telopeptide of type I collagen in post-menopausal women, thereby lowering bone turnover rates and ultimately reducing the incidence of OP (98). Moreover, combined lycopene and genistein treatment mitigates glucocorticoid-induced OP (99, 100). Taken together, lycopene supplementation represents a promising nutritional strategy for OP prevention, especially in post-menopausal women.

Other carotenoids also contribute to bone metabolism regulation. Tao et al. found that astaxanthin intervention significantly reversed the inhibitory effect of palmitate on osteogenic differentiation and the upregulation of osteoclast differentiation (101). At doses of 5 or 10 mg/kg, crocin not only prevents histopathological bone deterioration but also demonstrates a dual regulatory effect on bone turnover by elevating formation markers (ALP, OCN) and suppressing resorption markers (tartrate-resistant acid phosphatase and type I collagen cross-linked C-telopeptide) (102). Lutein, another carotenoid, effectively preserves bone mass by modulating both bone resorption and formation, showing potential for the prevention of disuse OP (103).

Carotenoids were also protective against 4-year bone density loss at the trochanter in men and at the lumbar spine in women (104). A Mendelian randomization study indicated that genetically predicted serum β-carotene levels are associated with increased BMD and a reduced risk of OP (105). Although β-carotene may contribute to higher bone density and a lower risk of OP and fractures, these effects may vary by sex and ethnicity (23).

#### 4.2.2 Antioxidant effects and bone health

Unlike β-carotene, lycopene lacks vitamin-A activity but is a powerful antioxidant that lowers the risk of age-related chronic disease (92). Clinical reports confirm that dietary antioxidants such as lycopene can decrease oxidative stress and bone turnover markers in post-menopausal women, contributing to a reduced risk of OP (91). Similarly, crocin reduces oxidative stress in distal femoral epiphyseal tissue and enhances the longitudinal and vertical mechanical properties of the femur, thereby improving metabolic syndrome-induced OP (102). The antioxidant properties of carotenoids may counteract mechanisms underlying OP related to cachexia (106).

The collective intake of antioxidant nutrients appears to lower the likelihood of OP in women (107). Dietary consumption of β-carotene and β-cryptoxanthin may benefit bone health (108), population-based studies indicate that, in combination with vitamin C, they are associated with a reduced risk of OP (108–110). And higher intakes of total carotenoids and lycopene are associated with reduced hip-fracture risk in long-term follow-up (111). In the European Prospective Investigation into Cancer and Nutrition (EPIC)–Norfolk cohort, dietary carotenoid intake was associated with improved bone health in both men and women, confirming that both intake and plasma concentrations of specific carotenoids correlate with BMD status and the risk of osteoporotic fractures (112). A prospective cohort study further demonstrated that antioxidant carotenoids—particularly β-cryptoxanthin and β-carotene—are inversely correlated with changes in radial BMD among post-menopausal women (113). Similarly, a study by Li-li Sun et al. indicated that dietary antioxidant nutrients, including β-carotene, are associated with a reduced risk of hip fracture among older Chinese adults (114). Total dietary carotenoids, as well as specific types such as α-carotene, β-carotene, and lutein/zeaxanthin, were negatively correlated with hip fracture risk. Furthermore, analysis of the National Health and Nutrition Examination Survey indicates that higher intakes of β-carotene, β-cryptoxanthin, lutein, and zeaxanthin are associated with reduced OP risk (115). In summary, dietary carotenoid intake is of considerable clinical relevance, as these antioxidants can reduce bone resorption, enhance BMD, and thereby help lower the risk of OP.

### 4.3 Regulatory effects of saponins on bone metabolism

Saponins are a class of natural compounds abundantly present in traditional Chinese medicines and have garnered significant interest for their potential role in the management of OP. Two decades ago, Nian et al. reported that anemarrhena steroidal saponins could prevent bone loss in ovariectomised rats (116). Subsequent research has further established the importance of saponins in the prevention and treatment of OP, with various types of saponins likely acting through shared mechanisms.

#### 4.3.1 Modulation of bone metabolism

On one hand, ginsenoside Rg3 influences osteoclast differentiation (117). Yanhuai Ma et al. observed that ginsenosides inhibit osteoclastogenesis and reduce bone loss in castrated mice, suggesting therapeutic potential for male OP (118). On the other hand, ginsenosides also regulate osteogenesis, offering novel targets and strategies for OP treatment (119). For instance, ginsenoside Rb1 promotes osteoblast differentiation and may hinder OP progression via modulation of the aryl hydrocarbon receptor (AHR)/Proline/arginine-rich end leucine-rich repeat protein (PRELP)/NF-κB axis (120). Ginsenoside Rb2 exhibits anti-osteoporotic effects by mitigating oxidative damage and osteoclast-related cytokines during bone formation (121). Network analysis revealed that ginsenoside Rh2 exhibits strong binding affinity to four target proteins (IL1β, TNF, IFNG, and NFKBIA), underscoring its relevance in OP treatment, with osteoblast differentiation emerging as a key signaling pathway (122). Moreover, ginsenoside Rg3 has been shown to alleviate aluminum-induced OP in rats by modulating oxidative stress, bone metabolism, and osteogenic activity (123, 124). Triterpenoid saponins from *Pimpinella candolleana* have been shown to promote osteogenic differentiation and improve trabecular bone structure, with efficacy comparable to that of alendronate (125). Furthermore, notoginsenosides inhibit radiation-induced OP by modulating the balance between bone formation and resorption (126).

#### 4.3.2 Anti-OP effects: preclinical evidence

The most extensively studied saponins are ginsenosides, which exert significant effects on osteoblasts, osteoclasts, and chondrocytes. They have demonstrated efficacy in increasing BMD and alleviating symptoms of osteoarthritis. Mechanistically, ginsenosides modulate cell differentiation, activity, and key signaling molecules such as MAPKs (127). Studies indicate that ginsenosides are beneficial across multiple forms of OP, including glucocorticoid-induced (119), castration-induced (118), ovariectomy-induced (128), and aluminum-induced models (123). Using network pharmacology, Zhang et al. identified five core gene clusters—STAT3, PIK3R1, VEGFA, JAK2, and MAP2K1—as potential therapeutic targets of ginsenosides in OP (129). It is noteworthy, however, that ginsenoside Rb1 did not prevent osteoporotic bone loss in ovariectomised rats (130). In summary, ginsenosides provide a robust theoretical foundation for future clinical applications in the treatment of OP (131).

Dipsacus asperoides is a traditional Chinese medicine with a history of over 2000 years in China. It is widely recognized for its ability to nourish the liver and kidneys, strengthen bones and muscles, and promote fracture healing. It is also commonly used in the treatment of OP, owing to the anti-osteoporotic effects of its saponin constituents (132). Guan et al. (133) isolated saponins from Dipsacus asperoides present in the absorbed components of the Xian-Ling-Gu-Bao capsule in the bloodstream and evaluated their anti-osteoporotic efficacy in a zebrafish model (133). The results demonstrated that these saponins could reverse prednisolone-induced reductions in bone mineralisation, confirming that they constitute the material basis for the pharmacological effects of the Xian-Ling-Gu-Bao capsule (133). Similarly, holothurian saponin A and holothurian glycoside A can increase bone density and bone deposition rates, reverse trabecular and bone marrow stromal loss, and thereby ameliorate ovariectomy-induced OP (134). Other saponins, such as Ziyu glycoside II (135), notoginsenosides (136), and soy saponins (137), have also demonstrated efficacy in alleviating OP in ovariectomised mice, making them promising natural candidates for the treatment of post-menopausal OP. Furthermore, astragaloside ASI-IV has been found to inhibit iron-load-induced bone loss in mice and protect against abnormal differentiation of BM-MSCs under iron overload conditions by regulating iron homeostasis and metabolism (138). Tu-bei-mu-gan-jia (tubeimoside I) shows protective effects against bone loss in rats with type 2 diabetes-induced OP (139). Anemarrhena saponin BII alleviates deterioration of the tibial microstructure in diabetic rats and reduces hyperglycaemia-induced apoptosis of primary rat calvarial osteoblasts in a dose-dependent manner (140) (Table 1).

**Table 1:** Plant-derived food bioactives and their anti-osteoporosis effects.

**Plant-derived food bioactives**	**Effects on OP**	**Representative substances**
Polyphenols	•Treating or preventing OP: • Chronic kidney disease-induced OP • Secondary OP in breast cancer patients • OP induced by high—altitude hypoxia • OP induced by male gonadal dysfunction • Rosiglitazone-induced OP in patients with type 2 diabetes • Senile and post-menopausal OP • Iron-overload-induced OP • Estrogen deficiency-induced OP •Antioxidant properties •Anti-inflammatory •Enhancing the abundance and functional activity of gut microbial communities	• Resveratrol • Curcumin • Quercetin • Flavonoids • Trifolirhizin • Polyphenol-rich extracts from *Calendula officinalis* (marigold) flowers • Danshensu • Polyphenols from areca nut seeds
Carotenoids	•Anti-OP effects: • Glucocorticoid-induced OP • Post-menopausal OP • Senile OP • OP related to cachexia • Disuse OP •Antioxidant properties •Promoting bone formation •Inhibiting bone resorption •Increasing BMD	• Lycopene • Astaxanthin • Crocin • Lutein • β-carotene • β-cryptoxanthin
Saponins	•Anti-OP effects: • Glucocorticoid-induced OP • Castration-induced OP • Aluminum-induced OP • Post-menopausal OP • Radiation-induced OP • Iron-load-induced • Type 2 diabetes-induced OP •Increasing BMD •Promoting bone formation •Inhibiting bone resorption •Regulating gut microbiota •Reducing inflammation	• Ginsenosides • Dipsacus asperoides • Triterpenoid saponins from *P. candolleana* • Holothurian saponin A • Ziyu glycoside II • Notoginsenosides • Soy saponins • Astragaloside • Tubeimoside I • Fenugreek-derived steroidal saponins

## 5 Regulatory mechanisms of major plant-derived bioactives on OP

### 5.1 Molecular mechanisms of polyphenols in regulating OP

The mechanisms by which phenolic compounds affect bone metabolism are complex. These mechanisms include stimulating the differentiation, maturation, and proliferation of osteoblasts through estrogen receptors, as well as activating key signaling pathways such as ERK 1/2 (141), p38 MAPK (142), and Wnt (143).

#### 5.1.1 Resveratrol exerts significant beneficial effects through multiple targets and pathways

Resveratrol regulates the balance between bone formation and resorption, promoting osteoblast differentiation via the PI3K/Akt, SIRT1, AMP-activated protein kinase (AMPK), and GATA binding protein 1 pathways while inhibiting osteoclastogenesis by suppressing MAPK and tumor necrosis factor receptor-associated factor 6/transforming growth factor-β-activated kinase 1(TRAF6/TAK1) signaling. This dual action helps maintain bone metabolic homeostasis and confers a bone-protective effect (67). For example, in a model of spinal cord injury-induced OP, Zhong (144) demonstrated that resveratrol enhances the bone-protective efficacy of calcium supplementation by modulating the SIRT1/forkhead box O3a (FOXO3a) pathway along with osteoblast and osteoclast activity. They proposed that the combination of resveratrol and calcium may represent an effective therapeutic strategy for the treatment of spinal cord injury-induced OP (144). At the molecular level, resveratrol inhibits osteoblast apoptosis by regulating proteins such as TNF, IL-6, and caspase-3, thereby promoting osteoblast formation (145). Furthermore, it counteracts the age-related decline in bone formation by improving osteogenic function in senescent BM-MSCs, an effect mediated through the enhancement of mitochondrial function via mitochondrial serine protease (146).

Antioxidant and anti-inflammatory mechanisms. Resveratrol mitigates oxidative stress and inflammation-induced bone loss through activation of the Hippo signaling pathway/Yes-associated protein (Hippo/YAP) and the Nrf2 pathway, as well as inhibition of the ROS/hypoxia-inducible factor-1α (ROS/HIF-1α) and nicotinamide adenine dinucleotide phosphate oxidase 4(Nox4)/NF-κB pathways (67, 147). Moreover, resveratrol enhances cellular resistance to oxidative damage and suppresses osteoclastogenesis by upregulating FoxO1 transcriptional activity via inhibition of the PI3K/Akt signaling pathway (148).

Targeted regulation in response to specific stimuli. Under hypoxic conditions, resveratrol enhances the osteogenic differentiation and mineralisation of BM-MSCs and upregulates osteogenic markers—including RUNX2, ALP, OCN, and OPN—through inhibition of the ROS/HIF-1α pathway (69). Notably, it ameliorates androgen-deficient bone loss by reestablishing the balance between RANK and OPG (70). Furthermore, resveratrol counteracts estrogen deficiency-induced OP through multiple mechanisms: it elevates miR-92b-3p expression to attenuate the NADPH oxidase 4/NF-κB pathway (149), inhibits miR-338-3p to upregulate Runx2 in osteoblasts (150), and facilitates osteoblast differentiation via SIRT1–NF-κB signaling (151).

#### 5.1.2 Diverse bone-protective mechanisms of other polyphenols

Activating Wnt/β-catenin signaling to promote osteogenesis. Polyphenol-rich extracts from *Calendula officinalis* (marigold) flowers (27) and anthocyanin-rich compounds derived from *Hibiscus sabdariffa* (hibiscus) petals (152) promote osteogenic differentiation and ameliorate osteoporotic bone loss by inhibiting glycogen synthase kinase 3β and subsequently activating β-catenin (27, 152). Berry polyphenols—anthocyanins in particular—also support bone health through antioxidant and anti-resorptive mechanisms (153). Additionally, danshensu attenuates bone marrow adiposity via the KLF15/peroxisome proliferator-activated receptor γ2(PPARγ2)/FOXO3a/Wnt axis, thereby counteracting glucocorticoid-induced OP (154).

Inhibiting osteoclastogenesis to suppress bone resorption. Trifolirhizin, a natural flavonoid glycoside, suppresses osteoclast differentiation and bone resorption through downregulation of key osteoclastogenic marker genes, signaling mediators, and bone resorption-associated proteins, alongside an increase in the serum OPG/RANKL ratio (90).

**Figure 2:**
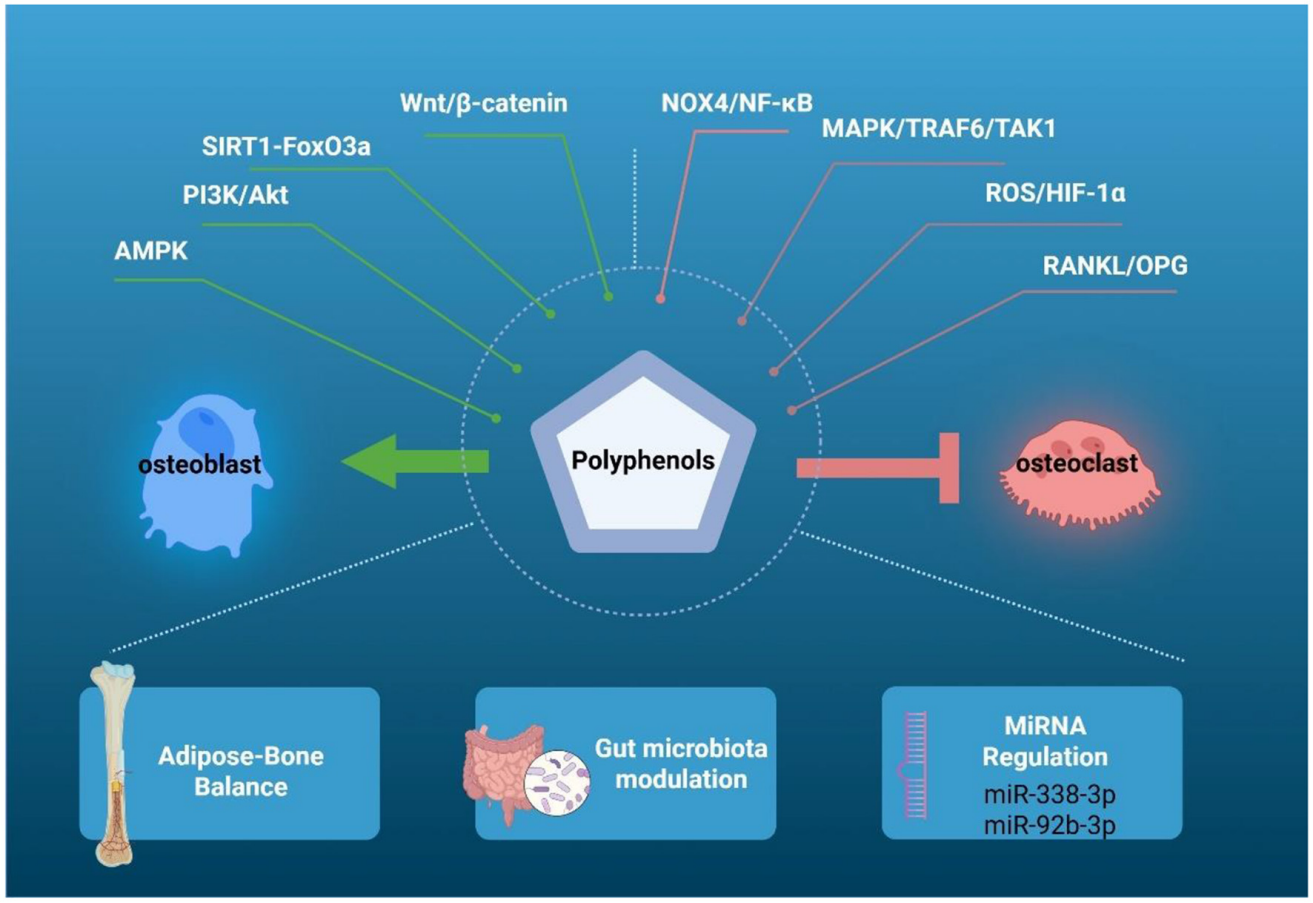
Mechanisms of polyphenols in OP prevention. Resveratrol, a polyphenol, promotes osteogenesis by activating PI3K/Akt, SIRT1, AMPK and Wnt pathways, and inhibits osteoclast differentiation and bone resorption by suppressing MAPK/TRAF6/TAK1 and lowering the RANKL/OPG ratio. It also inhibits ROS/HIF-1α and Nox4/NF-κB to reduce oxidative stress and bone loss. Resveratrol can inhibit miR-338-3p to upregulate the expression of runt-related transcription factor 2 in human osteoblasts, promoting osteogenesis, and upregulate miR-92b-3p to weaken the Nox4/NF-κB pathway, inhibiting bone resorption. Danshensu affects fat-bone balance and reduces marrow fat accumulation. Betel nut polyphenols regulate gut microbiota and improve osteoporosis by controlling inflammatory responses.

Modulating the Gut–Bone Axis and Bone Marrow Environment. Polyphenols from areca nut seeds enhance lysozyme expression by preserving Paneth cell populations, which correlates with gut microbiota modulation and amelioration of OP via control of inflammatory responses (155). Similarly, danshensu targets the adipose–bone metabolic crosstalk, reducing bone marrow fat accumulation and supporting bone integrity (154) (Figure 2).

### 5.2 Carotenoids mediate anti-osteoporotic effects through diverse molecular mechanisms

Lycopene mediates anti-osteoporotic activity through multiple mechanisms, with key pathways including the following: lycopene promotes the osteogenic differentiation of human BM-MSCs via the estrogen receptor 1 (ESR1)/PI3K/Akt signaling pathway, thereby counteracting bone loss and providing a molecular basis for its potential use in OP treatment (156). It also suppresses excessive ROS generation in the bone marrow and BM-MSCs of ovariectomised rats, reducing adipogenesis and favoring osteogenesis during bone remodeling (157). Furthermore, lycopene enhances osteoblastogenesis and inhibits adipogenesis by modulating the oxidative stress-driven FoxO1/PPARγ pathway, ameliorating osteoporotic bone loss (157). Lycopene may also mitigate age-related bone deterioration by inhibiting oxidative stress, cellular senescence, and the SASP, supporting its role in the management of senile OP (158). Additionally, the combination of lycopene with genistein—particularly when co-administered—may counteract glucocorticoid-induced adverse effects through complementary molecular pathways such as Wnt/β-catenin and Nrf2, thereby stimulating bone formation, reducing bone resorption, and improving bone architecture (100).

Astaxanthin may inhibit palmitate-induced bone loss via its antioxidant properties, mediated through the SIRT1 signaling pathway (101). It also specifically targets osteoclasts: Hwang et al. demonstrated that astaxanthin suppresses osteoclast formation by regulating the expression of NFATc1, dendritic cell-specific transmembrane protein, tartrate-resistant acid phosphatase, and CTSK, without exerting cytotoxic effects on bone marrow-derived macrophages, indicating therapeutic potential for post-menopausal OP (159). Lutein plays a key role in protecting ovariectomised rats from OP through activation of Nrf2 and downregulation of inflammatory responses and osteoclast-specific markers, including NFATc1 (160).

### 5.3 Molecular mechanisms of saponins in OP

The molecular mechanisms through which saponins exert their preventive and therapeutic effects on OP primarily encompass the following aspects:

#### 5.3.1 PI3K/Akt/mTOR signaling pathway

Ginsenosides may modulate osteogenesis by mediating the expression of the G protein-coupled estrogen receptor, which in turn modulates Akt phosphorylation within the PI3K/Akt pathway (119). Zhang et al. also reported that ginsenoside Rg3 attenuates OP induced by ovariectomy via the AMPK/mTOR signaling pathway (161). Similarly, dipsacoside VI promotes osteogenic differentiation of bone marrow stromal cells in ovariectomised rats through the PI3K/Akt signaling pathway (162). Furthermore, notoginsenosides have been shown to regulate the expression of angiogenesis-related factors via the PI3K/Akt/mTOR pathway, thereby facilitating the healing of osteoporotic fractures in ovariectomised rats (163). Additionally, anemarrhena saponin BII activates autophagy in osteoblasts by inhibiting the mTOR/NF-κB pathway, thus ameliorating high glucose-induced oxidative stress and apoptosis (140).

#### 5.3.2 BMP-2/BMPR1A/Runx2 axis

Zhang et al. (164) demonstrated that ginsenoside Rg3 mitigates glucocorticoid-induced OP by regulating the BMP-2/BMPR1A/Runx2 signaling pathway (164). Astragaloside IV also promotes osteogenic differentiation of BM-MSCs through the miR-21/nerve growth factor/BMP2/Runx2 pathway (165). Moreover, soyasaponin Bb, present in peanut sprouts, enhances the expression of the osteogenic transcription factor Runx2 and ALP, showing potential for the prevention of bone disorders, including OP (166).

#### 5.3.3 RANKL/RANK/OPG signaling pathway

Triterpenoid saponins from *P. candolleana* activate the P38/c-Jun N-terminal kinase (JNK) MAPK pathway and upregulate the OPG/RANKL axis, thereby modulating bone metabolism and stimulating osteogenesis (125). Fenugreek-derived steroidal saponins inhibit the colony-stimulating factor 1 (CSF-1)/CSF-1R-induced phosphorylation signaling pathway in both osteoclasts and osteoblasts. This results in suppression of RANK expression in osteoclasts and reduction of ROS generation in osteoblasts. Consequently, the ratio of RANKL to OPG is decreased, leading to diminished survival, proliferation, and differentiation of osteoclasts (167).

#### 5.3.4 MAPK signaling pathway

Ginsenosides modulate osteoclast differentiation through the c-Fms-mediated MAPK and PI3K signaling axis (129). Platycodon saponin D, the most abundant and pharmacologically active triterpenoid saponin in Platycodon grandiflorum, inhibits RANKL-induced activation of NF-κB, ERK, and p38 MAPK, ultimately suppressing osteoclast differentiation (168). Similarly, bupleurum saponin A inhibits osteoclastogenesis by attenuating RANKL-induced activation of p38, ERK, JNK, and NF-κB, demonstrating potential as a novel therapeutic agent for OP (169).

#### 5.3.5 NF-κB signaling pathway

Studies show that ginsenosides exert anti-osteoporotic effects chiefly by suppressing osteoclastogenesis through the NF-κB/MAPK signaling pathway (117, 118, 170). Astragaloside IV may inhibit macrophage senescence and stimulate the osteogenic differentiation of BM-MSCs by modulating the stimulator of interferon genes/NF-κB pathway, thereby exerting anti-osteoporotic effects. Consequently, astragaloside IV shows promise as a therapeutic candidate for the treatment of OP (171). In addition, holothurin A and echinoside A were found to significantly downregulate the expression of inhibitor of kappa B kinase, NF-κB, and phosphorylated NF-κB p65, and inhibit the expression of the osteoclastogenic transcription factors c-Fos and NFATc1 (134). Furthermore, tubeimoside I also ameliorates bone loss in rats with type 2 diabetic osteoporosis in the same manner (139).

#### 5.3.6 Others

**Figure 3:**
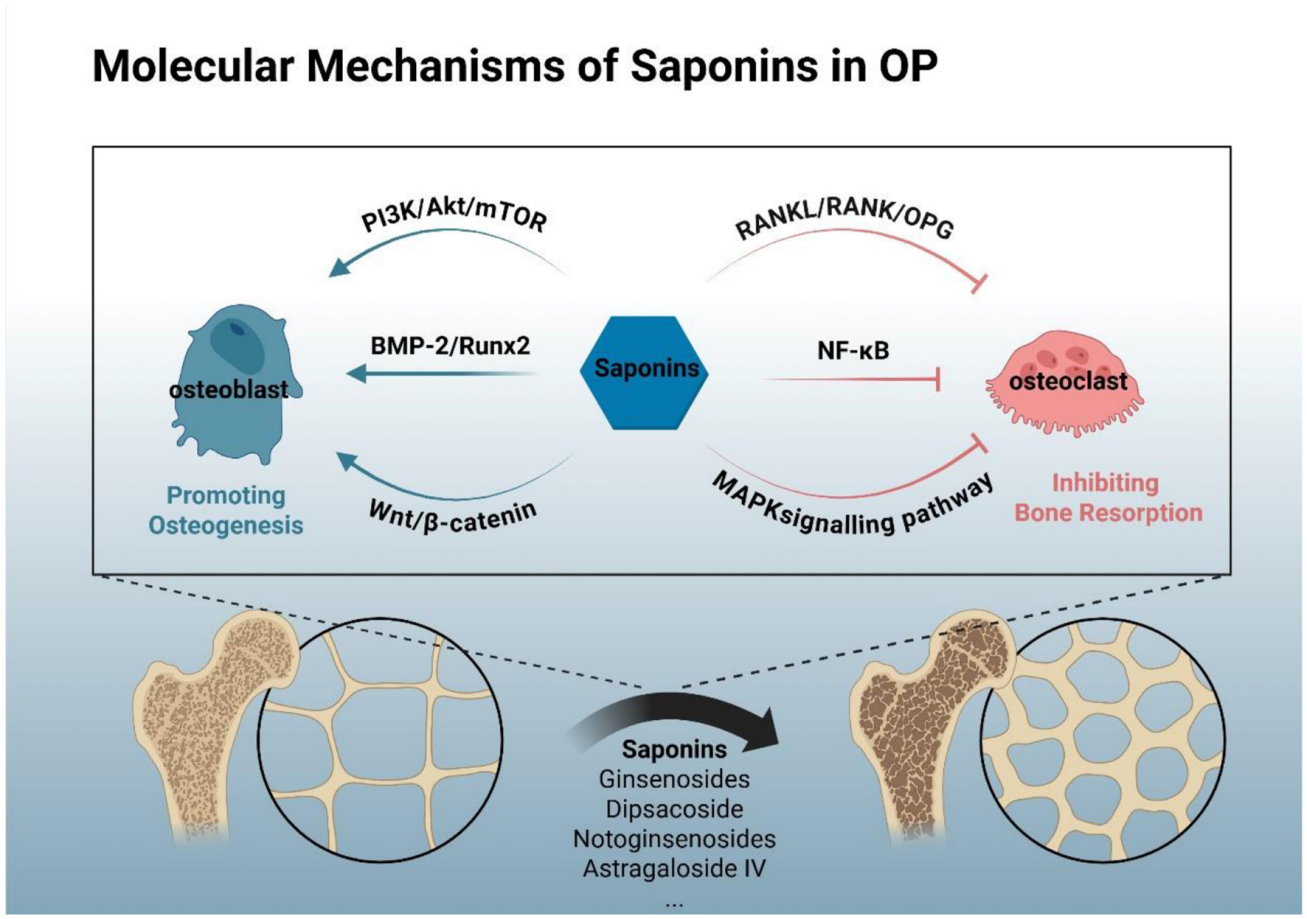
Molecular mechanisms of saponins in osteoporosis prevention. (SaponinsGinsenosides, Dipsacoside, notoginsenosides, Astragaloside IV, etc.) Reshape the balance of bone metabolism by promoting osteogenesis and inhibiting osteoclastogenesis, thereby treating osteoporosis. Saponins mainly promote osteogenesis through the PI3K/Akt/mTOR, BMP-2/Runx2 axis, Wnt/β-catenin and other signaling pathways, and inhibit osteoclast formation and reduce bone resorption through the RANKL/RANK/OPG, MAPK, NF-κB signaling pathways.

Ginsenoside Rc promotes bone formation in ovariectomised mice-induced OP *in vivo* and osteogenic differentiation *in vitro* via the Wnt/β-catenin signaling pathway (128). Another study has shown that ginsenoside Rg1 modulates pathways principally involved in retinol, fat, protein and lipid metabolism, a mechanism that may underlie its ability to counter glucocorticoid-induced osteoporosis (172). Astragaloside IV (AST-IV) can promote the differentiation of BM-MSCs, with glycogen synthase kinase 3β signaling pathway involved in its osteogenesis induction, and it accelerates cell differentiation by increasing the expression level of nerve growth factor (173). Ziyu Glycoside II can also alleviate bone loss in ovariectomised mice by reducing inflammation, regulating gut microbiota (including unclassified *Muribaculaceae* family and *Dysgonomonas* genus) and short-chain fatty acids (135). Hu et al. (136) found through ovariectomised mice that notoginsenosides can activate osteogenesis and angiogenesis, thereby increasing bone mass, indicating its potential role in the prevention and treatment of OP in post-menopausal women (136). Furthermore, a study has shown that asperosaponin VI restores expression of the anti-ferroptotic factor GPX4, thereby attenuating the ferroptotic pathology linked to diabetic osteoporosis and positioning it as a potential therapeutic agent for this complication (174) (Figure 3).

## 6 Influence of experimental models on evaluating bioactive efficacy: a comparative perspective

The preclinical evidence presented here derives largely from distinct animal models of OP, each recapitulating specific etiologies. A critical appraisal of how these model-specific pathophysiologies shape both the observed efficacy and the dominant mechanisms of plant-derived bioactives is essential if such findings are to be translated into targeted clinical contexts. Here we compare the two most widely used paradigms: the ovariectomy model, which mimics post-menopausal OP, and the glucocorticoid-induced model, representing secondary OP.

### 6.1 Pathophysiological dichotomy: ovariectomy model vs. glucocorticoid-induced models

The ovariectomy model is driven chiefly by estrogen deficiency, producing high-turnover bone loss in which osteoclastic resorption is markedly accelerated. Key molecular hallmarks include a significantly elevated RANKL/OPG ratio, enhanced NF-κB/MAPK signaling in osteoclast precursors and a pro-inflammatory microenvironment (175). By contrast, the glucocorticoid-induced model suppresses osteoblast activity directly and promotes apoptosis of osteoblasts and osteocytes, yielding a low-formation lesion (176). Although resorption may rise initially, the dominant defect is inhibition of anabolic pathways—principally the Wnt/β-catenin and BMP-2/Runx2 axes.

### 6.2 Consequences for bioactive efficacy and mechanism

The dichotomy described above determines how plant-derived bioactives manifest their polyvalent actions (Table 2). In the high-turnover ovariectomy model, compounds that curb resorption and inflammation are expected to excel: polyphenols such as resveratrol suppress RANKL signaling, NF-κB activation and osteoclast-specific transcription factors (NFATc1, c-Fos), thereby directly opposing the principal driver of estrogen-deficient bone loss, an effect consistent with the protection afforded by resveratrol and trifolirhizin in numerous ovariectomy studies (76, 84, 90). Likewise, saponins—ginsenoside Rb2 and fenugreek saponins, for example—that inhibit the RANKL/OPG/NF-κB/MAPK axis effectively blunt the exaggerated osteoclastogenesis of this model (118, 167). Conversely, the low-formation glucocorticoid-induced model favors agents that enhance anabolism and safeguard osteoblasts: the lycopene–genistein combination stimulates Wnt/β-catenin and Nrf2 signaling, directly countering the suppressed osteoblastogenesis and oxidative stress characteristic of glucocorticoid exposure (100), while saponins such as ginsenoside Rg3 and astragaloside IV, which up-regulate the BMP-2/Runx2 axis and promote marrow stromal-cell differentiation, offer a mechanistic remedy for impaired bone formation (164, 165). Divergent outcomes across models reinforce the point: ginsenoside Rb1, effective in a glucocorticoid-relevant AHR/PRELP/NF-κB pathway (120), is without benefit in ovariectomised rats (130), indicating that its action aligns with correction of glucocorticoid-induced dysregulation rather than estrogen-deficiency-driven hyper-resorption, and the deliberate tailoring of the lycopene–genistein combination to glucocorticoid-induced pathology underlines the need for model-guided selection of bioactives.

**Table 2:** Comparative analysis of principal OP models and their implications for the evaluation of plant-derived bioactives.

**Feature**	**Ovariectomy model (post-menopausal OP)**	**Glucocorticoid-induced model (glucocorticoid-induced OP)**
Primary induction	Surgical removal of ovaries (estrogen deficiency)	Administration of high-dose glucocorticoids
Core pathology	High bone turnover; Dominant increase in bone resorption	Low bone formation; Dominant suppression of osteoblast function
Key molecular hallmarks	↑RANKL/OPG ratio; ↑NF-κB/MAPK signaling in osteoclasts; Pro-inflammatory state	↓ Wnt/β-catenin signaling; ↓ BMP-2/Runx2 activity; ↑ Osteoblast/osteocyte apoptosis
Ideal for evaluating	Anti-resorptive and anti-inflammatory mechanisms	Anabolic (pro-osteogenic) and osteoblast/osteocyte-protective mechanisms
Relevant bioactives	• Polyphenols: Resveratrol, Trifolirhizin (inhibit RANKL/NF-κB) • Carotenoids: Astaxanthin, Lutein, lycopene • Saponins: Ginsenoside Rb2, Notoginsenosides, saponin A, Ziyu glycoside II, soy saponins, Fenugreek saponins (suppress osteoclastogenesis)	• Polyphenols: danshensu • Carotenoids: Lycopene + Genistein combo (activate Wnt/Nrf2) • Saponins: Ginsenoside Rg3 (enhances BMP-2/Runx2), Astragaloside IV (promotes osteogenesis)
Clinical translation context	Prevention and management of post-menopausal OP	Prevention of secondary osteoporosis in patients on long-term corticosteroid therapy

The inherent “model bias” within pre-clinical evidence is not a limitation, but rather a prerequisite for precision. Data from ovariectomised animals provide robust support for deploying anti-resorptive bioactives—selected polyphenols or saponins—in the management of post-menopausal OP, whereas glucocorticoid-based studies argue equally forcefully for pro-osteogenic formulations such as carotenoid–isoflavone combinations or specific saponins to forestall glucocorticoid-induced bone loss. Such a comparative framework will accelerate the formulation of etiology-specific nutraceutical strategies for OP.

## 7 Summary and outlook

This review summarizes the roles and mechanisms of dietary bioactive compounds—including polyphenols, carotenoids, and saponins—in the regulation of OP, with a particular emphasis on their core ability to modulate bone metabolism through coordinated multi-pathway actions. These bioactive substances counteract osteoporosis via a triple mechanism centered on antioxidant, anti-inflammatory, and bone metabolism-regulating properties. Polyphenols (e.g., resveratrol) activate the SIRT1/FOXO3a, Hippo/YAP, and Nrf2 pathways to enhance osteogenic differentiation, while inhibiting the ROS/HIF-1α and Nox4/NF-κB pathways to attenuate oxidative stress and inflammation (67, 69, 160). They also modulate the gut–bone axis (e.g., by elevating levels of the metabolite 4-HPA) and suppress osteoclast-related genes such as NFATc1 and CTSK (63). Carotenoids (e.g., lycopene and astaxanthin) promote osteogenesis through the ESR1/PI3K/Akt and FoxO1/PPARγ pathways (156, 157), inhibit adipogenesis via Nrf-2-mediated reduction of ROS accumulation, and impede osteoclast maturation by targeting markers including tartrate-resistant acid phosphatase and dendritic cell-specific transmembrane protein (100, 159). Saponins (e.g., ginsenosides and dipsacosides) regulate bone remodeling bidirectionally via pathways such as PI3K/Akt/mTOR, BMP-2/Runx2, and RANKL/OPG (163, 164, 167). They also inhibit NF-κB/MAPK signaling, leading to the downregulation of osteoclast-related transcription factors such as c-Fos and NFATc1 (170). Dietary bioactive compounds show considerable promise for clinical application in the prevention and treatment of osteoporosis. They offer notable safety advantages over synthetic pharmaceuticals, avoiding risks such as hypercalcaemia and thromboembolism associated with conventional treatments (e.g., bisphosphonates). Moreover, combination therapies may enhance efficacy: for example, resveratrol combined with calcium improves bone density in individuals with spinal cord injury, while lycopene and genistein act synergistically to ameliorate glucocorticoid-induced bone loss.

Promising pre-clinical findings for plant-derived bioactives must be set against a number of fundamental obstacles that presently hinder their direct translation to the clinic. First, poor systemic bioavailability markedly restricts the efficacy of many compounds—most polyphenols, for example—because their active forms may not reach bone tissue at adequate concentrations (63). Second, the non-linear, multi-target mode of action complicates the establishment of clear dose–effect relationships, making it difficult to define optimal therapeutic windows when moving from animal studies to human trials. Third, considerable inter-individual variability, especially in gut-microbiota composition and function, governs the metabolism and activation of many food bioactives and, ultimately, their biological effects (e.g., the conversion of polyphenols to active metabolites such as 4-HPA) (77, 78). Such variability presents a major barrier to achieving consistent clinical outcomes. Moreover, many current studies are confined to *in vitro* cellular experiments or animal models—such as ovariectomised rats and glucocorticoid-induced mice—and lack large-scale, multicentre, long-term follow-up clinical trials in humans. Certain clinical studies that do exist often involve small sample sizes and short follow-up periods, making it difficult to draw definitive conclusions regarding long-term effects on BMD and fracture risk. Thus, while multi-target activity is a strength, it simultaneously introduces complexity in standardization and in the reproducible assessment of efficacy.

Although pre-clinical studies have shown promise, the translation of plant-derived food bioactives into interventions for OP remains problematic. To realize their therapeutic potential, future work must move beyond descriptive validation and embrace mechanistically targeted investigation. Organoid and organ-on-a-chip technologies offer unprecedented investigative opportunities. By co-culturing intestinal organoids with marrow-derived osteoblasts or osteoclast precursors on microfluidic chips, a tractable “gut–bone axis” can be engineered (55). Such systems permit real-time examination, within a physiologically relevant microenvironment, of how specific bioactive metabolites—such as the polyphenol derivative 4-HPA (63)—or shifts in the microbial community directly modulate bone-cell behavior.

Future investigations should stratify participants on the basis of predictive biomarkers. These may comprise: (i) gut-microbiome profiles, such as the baseline abundance of polyphenol-metabolizing bacteria (78); (ii) metabolomic signatures, including levels of short-chain fatty acids or inflammatory metabolites (54); and (iii) genetic or epigenetic markers, for example polymorphisms in antioxidant-response or estrogen-signaling pathways. Interventions can then be tailored accordingly, and their efficacy evaluated against these biomarkers in conjunction with BMD.

Owing to the polygenic and multi-target nature of both OP pathogenesis and bioactive action, future work should systematically identify synergistic combinations. Examples already hint at benefit: resveratrol plus calcium/vitamin D mitigates spinal-cord-injury-induced bone loss (144), whereas lycopene combined with genistein is markedly protective in glucocorticoid-driven OP (100). Resveratrol-loaded hydrogels enhance osteogenic differentiation (177). Likewise, co-formulation of polyphenols with probiotics is being explored to enhance bioavailability and gut-mediated effects (80). Network-pharmacology and systems-biology frameworks should now be deployed to rationally optimize such combinations (122, 129).

Plant-derived food bioactives constitute a large and promising arsenal for the prevention and management of OP, characterized by multi-target activity and favorable safety profiles. The way forward is to adopt innovative models, design smarter clinical trials, apply cutting-edge technologies to dissect mechanisms, and develop engineered solutions to overcome bioavailability hurdles. By pursuing these targeted, interdisciplinary lines of research, we can accelerate the transition of these natural compounds from generic dietary constituents to validated, precise, and effective nutraceutical agents for bone health.
